# Antitoxic Serum in Tetanus

**Published:** 1893-08-12

**Authors:** 


### NEW DRUGS AND PREPARATIONS.

[We should be glad to receive, at our office, 428, Strand, London, W.C., from the manufacturers, specimens of all new preparations and drugs which may be brought out from time to time.]

### ANTITOXIC SERUM IN TETANUS

After the discovery of the tetanus bacillus in 1884, and the separation of its toxine by Kitasato, various attempts were made to find a remedy which should be really efficient against it. Behring and others have succeeded not only in rendering animals immune against the disease, but also in curing those affected by injecting the serum of protected animals. Immunity was produced by progressively increased injections of the toxine, and the serum of the animals thus healed is found to confer like immunity on others when injected in the proportion of 1 to 1,000,000 of the body weight. Behring determined that to cure the disease when it had actually commenced, the amount of serum employed must be increased to one in a thousand of the body weight, and even then it must be given early during certain stages of the disorder. Under these conditions it was found possible to attain very successful results.

Thus sheep suffering from tetanus were completely cured by the injection of 50 grammes of the serum of an immune horse. Since then a number of men who had been attacked by the disease have been inoculated with similar serum, or by a dried alcoholic extract of it. Roux and Vaillard in a recent review of all the reported cases and their results in the "Annales de l'Institute Pasteur," conclude that the injection of antitoxic serum is the only rational treatment, that it is without danger, and that it actually destroys the poison generated in the wound. They advise that when tetanus is diagnosed 100 cubic centimetres of antitoxic serum should be at once administered hypodermically, and the wound should be scraped out. The injections are to be repeated on the second, third, and tenth days.

One of the first cases was that of a groom in Holland, treated by Dr. Rotter, with immune horse serum, mixed with ·5 per cent. of carbolic acid. On successive days 66, 50, 45, and 50 centigrammes were injected, the symptoms gradually subsided, and complete recovery followed. The injections were made in four places in the back.

Ten other cases were treated by a preparation of the alcoholic extract under the care of Drs. Tizzoni and Cattani of Bologna. All of them likewise recovered, but it has been remarked that none of them were of a severe type, and that therefore the effect of the serum was not absolutely certain. Indeed, Bacelli insists that the carbolic acid added to the extract was really the active agent, and both he and Bertini report cases which have been treated by carbolic acid alone. One of Finotti's recent cases was remarkable. A youth of 19 years was wounded in the neck by a splinter in August. Part of the splinter was left in, and the wound seemed to be healing badly. On September 9th tetanus set in. The wound was then reopened, part of the splinter found and removed. The wound was excised and cauterised. The secretions from it produced tetanus when inoculated into animals. An injection of ·25 grammes of antitoxin was given on September 12th, two similar ones on the 13th, and one on the 14th." Under this treatment the patient rapidly improved, but the supply of serum ran short, and the spasms returned, the patient becoming much worse. From the 20th to the 27th seven more injections were administered with rapidly successful results. The symptoms passed away, and on October 3rd the patient was completely well. In a case of Schanz reported in the *British Medical Journal*, Jan. 2nd, 1892, 20 centigrammes of antitoxin were injected daily for five days. Pacini used 25 centigrammes twice daily for sixteen days, with occasional doses of choral, and Casili the same amount for three days. The success appears to depend, amongst other things, on the stage at which the treatment is commenced. In two cases under Renon the treatment was probably useless from this cause.

Taruffi treated an old man of seventy-four with injections of 25 centigrammes once and sometimes twice a day for eleven days with complete success.

Albertini thinks that the thorough local treatment which most of these underwent when antitoxins were employed had a great deal to do with the result. He pointed out that in 176 cases treated by various other methods 139 were reported as cured, and that therefore the disease is less fatal than is commonly supposed.

Roux and Vaillard believe that success depends largely on the amount of serum used and the quickness with which it is given after infection. If a certain time is allowed to elapse the bacilli multiply, and then manufacture their toxin in such quantity that a cure is impossible. Hence, too, the importance of clearing out the colony of germs by excision as far as possible, and of administering the antitoxin upon the first suspicion of infection. It may be discussed how far the remedy actually destroys the bacilli or merely neutralises their poison. If the latter is the case, then the body cells have time given to destroy the bacilli; and, at any rate, if the patient's life is preserved for a sufficient time, the disease tends to a natural cure. In view of the terrible effects and the frequent deaths produced by tetanus, this method of treatment cannot be neglected, and will doubtless be put to frequent tests in the future.

